# The COX-2/PGI2 Receptor Axis Plays an Obligatory Role in Mediating the Cardioprotection Conferred by the Late Phase of Ischemic Preconditioning

**DOI:** 10.1371/journal.pone.0041178

**Published:** 2012-07-23

**Authors:** Yiru Guo, Deepali Nivas Tukaye, Wen-Jian Wu, Xiaoping Zhu, Michael Book, Wei Tan, Steven P. Jones, Gregg Rokosh, Shuh Narumiya, Qianhong Li, Roberto Bolli

**Affiliations:** 1 Institute of Molecular Cardiology, University of Louisville, Louisville, Kentucky, United States of America; 2 Department of Internal Medicine, University of Louisville, Louisville, Kentucky, United States of America; 3 Department of Pharmacology, Kyoto University Faculty of Medicine, Kyoto, Japan; Virginia Commonwealth University Medical Center, United States of America

## Abstract

**Background:**

Pharmacologic studies with cyclooxygenase-2 (COX-2) inhibitors suggest that the late phase of ischemic preconditioning (PC) is mediated by COX-2. However, nonspecific effects of COX-2 inhibitors cannot be ruled out, and the selectivity of these inhibitors for COX-2 vs. COX-1 is only relative. Furthermore, the specific prostaglandin (PG) receptors responsible for the salubrious actions of COX-2-derived prostanoids remain unclear.

**Objective:**

To determine the role of COX-2 and prostacyclin receptor (IP) in late PC by gene deletion.

**Methods:**

COX-2 knockout (KO) mice (COX-2^−/−^), prostacyclin receptor KO (IP^−/−^) mice, and respective wildtype (WT, COX-2^+/+^ and IP^+/+^) mice underwent sham surgery or PC with six 4-min coronary occlusion (O)/4-min R cycles 24 h before a 30-min O/24 h R.

**Results:**

There were no significant differences in infarct size (IS) between non-preconditioned (non-PC) COX-2^+/+^, COX-2^−/−^, IP^+/+^, and IP^−/−^ mice, indicating that neither COX-2 nor IP modulates IS in the absence of PC. When COX-2^−/−^ or IP^−/−^ mice were preconditioned, IS was not reduced, indicating that the protection of late PC was completely abrogated by deletion of either the COX-2 or the IP gene. Administration of the IP selective antagonist, RO3244794 to C57BL6/J (B6) mice 30 min prior to the 30-min O had no effect on IS. When B6 mice were preconditioned 24 h prior to the 30-min O, IS was markedly reduced; however, the protection of late PC was completely abrogated by pretreatment of RO3244794.

**Conclusions:**

This is the first study to demonstrate that targeted disruption of the COX-2 gene completely abrogates the infarct-sparing effect of late PC, and that the IP, downstream of the COX-2/prostanoid pathway, is a key mediator of the late PC. These results provide unequivocal molecular genetic evidence for an essential role of the COX-2/PGI2 receptor axis in the cardioprotection afforded by the late PC.

## Introduction

The cardioprotective effect afforded by late PC is a well-documented and studied phenomenon [1]–[6]. In the last two decades, extensive research has identified the molecular candidates involved in late PC [7]. Among the numerous identified players, nitric oxide synthase [8]–[19], heat shock protein [20]–[23], Mn-superoxide dismutase [24], [25], extracellular superoxide dismutase [26], [27], aldose reductase [28] and COX-2 [15], [18], [29]–[47] are candidates for pharmacological modulation with the goal of developing cardioprotective therapies.

Previous studies have shown that COX-2 mediates its effects via increasing the synthesis of prostaglandin E2 (PGE2) and prostacyclin (PGI2) [29], [36]. The identification of specific molecules involved in the late phase of PC provides a unique opportunity to develop targeted therapy to exploit the phenomenon of PC for cardioprotection.

Our current knowledge about the role of COX-2 in the late phase of PC is based on pharmacologic studies with COX-2 inhibitors [29]–[31], [35]–[38], [41], [43], [46]–[48]. The possible nonspecific nature of COX-2 inhibitors raises the possibility that the observed inhibition of the late phase of PC may be secondary to non-specific inhibition of other molecules including COX-1 [49]. Furthermore, the specific downstream molecules transducing the actions of COX-2/prostanoids in late PC are unclear. Earlier studies have indicated that the prostacyclin receptor, IP, confers tissue protection [50]–[55]. In the present study, we examined the effect on late PC of homozygous COX-2 deletion; in addition, we explored the role of the prostaglandin receptor, espicailly IP, as a downstream mediator of COX-2 in late PC using both pharmacological and genetic approaches to manipulate IP gene function. Our results demonstrate the obligatory role of COX-2 in late PC by genetically deleting COX-2, thereby unequivocally establishing COX-2 as a mediator of the late phase of PC. In addition, we demonstrate an essential role of IP in mediating the cardioprotective effects of the late phase of PC.

**Figure 1:**
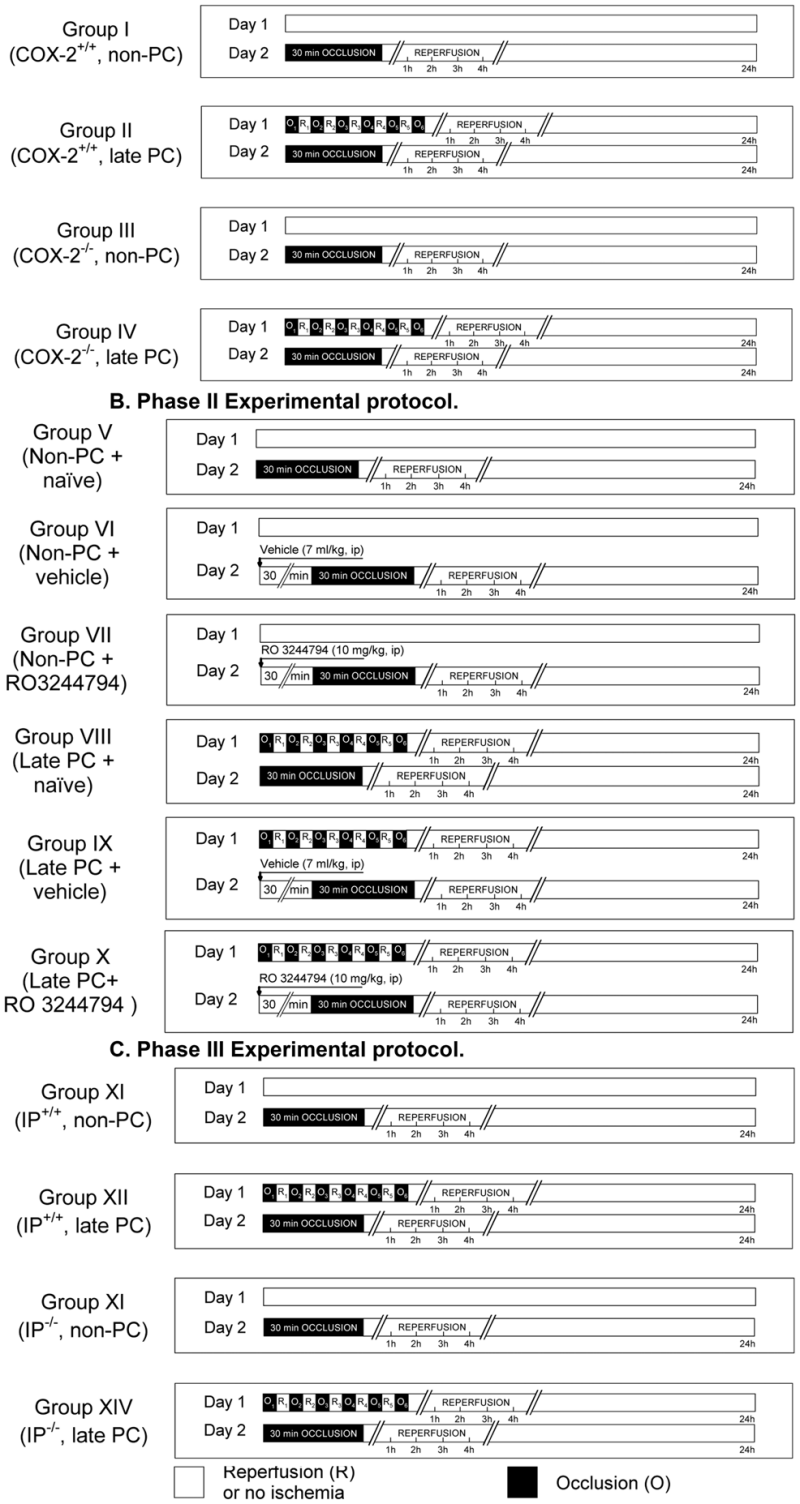
Experimental protocols. Fourteen groups of mice including were studied for infarct size analysis in three phases. In **Phase I** (**panel A**), on day1, *COX-2^+/+^* and *COX-2^−/−^* mice were subjected to either PC or sham surgery. On day 2, all mice were subjected to a 30-min LAD occlusion followed by 24 h of reperfusion. In **Phase II** (**panel B**), in addition to the day 2 protocol of Phase I, RO3244794 or vehicle was administered 30 min prior to the induction of acute MI on day 2. In **Phase III** (**panel C**), on day 1, *IP^+/+^* and *IP^−/−^* mice were subjected either to PC or sham surgery. On day 2, all mice were subjected to a 30-min LAD occlusion followed by 24 h of reperfusion. All animals were sacrificed after 24 h of reperfusion to measure infarct size. The open square (□) indicates the reperfusion or no ischemia protocol. The solid black square (▪) indicates the occlusion protocol. (n = 6–16 each group).

## Materials and Methods

This study was performed in accordance with the guidelines and with approval of the Institutional Animal Care and Use Committee at the University of Louisville, and with the *Guide for the Care and Use of Laboratory Animals* (Department of Health and Human Services, National Institutes of Health, Publication No. 86-23, revised 1996).

### Reagents

1. RO3244794 (R-3-(4-fluoro-phenyl)-2-[5-(4-fluoro-phenyl)-benzofuran-2-ylmethoxycarbonylamino]-propionicacid) was obtained from Roche Alto (Roche Palo Alto, CA). RO3244794 was solubilized in 0.2 M Trizma base which served as the vehicle [56], [57]; 2. Iloprost, (Cayman Chemical Co., Ann Arbor, MI); 3. Krebs-Henseleit Buffer Modified solution (Sigma-Aldrich Corp., St. Louis, MO USA); 4. TTC (Sigma-Aldrich Corp. St. Louis, MO USA); 5. Phthalo blue (Heucotech, Fairless Hill, PA).

**Table 1:** Reasons for excluding mice from study (15 groups).

Group	*I*	*II*	*III*	*IV*	*V*	*VI*	*VII*	*VIII*	*IX*	*XI*	*XI*	*XII*	*XIII*	*XIV*	*Pilot*	Total
**Bleeding**	0	0	1	1	0	0	0	0	0	0	0	0	0	0	0	2
**Death**	1	2	4	5	2	0	0	2	2	0	1	1	1	5	0	26
**Technical problems**	2	2	2	1	2	0	0	0	1	1	0	1	0	0	0	12
**Poor postmortem staining**	0	1	0	1	0	0	0	1	0	0	0	0	0	0	0	3
**Mice instrumented**	16	20	18	19	19	6	8	12	13	9	17	15	13	17	9	211
**Mice excluded**	3	5	7	8	4	0	0	3	3	1	1	2	1	5	0	43
**Mortality rate (%)**	6	10	22	26	11	0	0	17	15	0	6	7	8	29	0	12
**Mice included in study**	13	15	11	11	15	6	8	9	10	8	16	13	12	12	9	168
**Mice included in study (%)**	81	75	65	58	79	100	100	75	77	89	94	87	92	71	100	80

I, *COX-2^+/+^*, non-PC group; II, *COX-2^+/+^*, late PC group; III, *COX-2^−/−^*, non-PC group; IV, *COX-2^−/−^*, late PC group; V, B6, non-PC group; VI, B6, non-PC + vehicle group; VII, B6, non-PC + RO3244794 group; VIII, B6, late PC group; IX, B6, late PC + vehicle group; X, B6, late PC + RO3244794 group; XI, *IP^+/+^*, non-PC group; XII, *IP^+/+^*, late PC group; XIII, *IP^−/−^*, non-PC group; XIV, *IP^−/−^*, late PC group; Pilot, animals used for hemodynamic studies with RO3244794 and iloprost.

### Mice

Male mice were used in this study. The COX-2 knockout (COX-2^−/−^) and wildtype (COX-2^+/+^) mice [58] were generously provided by Dr. Robert Langenbach (NIEHS, NIH, NC). Their genetic background was 129Ola/C57BL/6. RO3244794 selective IP inhibition studies were performed in male C57BL6/J (B6) mice. Heterozygous IP KO breeding pairs [59] were provided by Dr. Shuh Narumiya (Tokyo University). We used male wildtype littermates (*IP^+/+^*) as control mice and homozygous IP KO (*IP^−/−^*). PCR and Southern blot hybridization were used for genotyping.

### Hemodynamic Pilot Study

To verify the specificity and dosage of specific IP antagonist RO3244794, we monitored arterial blood pressure during the administration of the specific IP agonist, iloprost (30 µg/kg, iv) with either vehicle or RO3244794 to see whether the hypotensive effect induced by iloprost could be prevented. This study was also conducted using *IP^−/−^* mice. In selected pilot studies, a catheter was inserted into the carotid artery for measurement of blood pressure (DTXTM pressure transducer, Viggo-Spectramed, Oxnard, CA). Surface leads were placed subcutaneously to obtain the ECG, which was recorded throughout the experiments on a thermal array chart recorder (Gould TA6000) [1], [9], [30], [60].

### Preconditioning (PC) and Myocardial Infarction *in vivo* Protocols

The murine model of late PC has been previously described in detail [1], [9], [17], [30], [61], [62]. Briefly, on day 1, mice were anesthetized with sodium pentobarbital (60 mg/kg, i.p), intubated, and ventilated with room air supplemented with oxygen at a rate of 105 strokes/min and with a tidal volume of 0.3±0.1 ml using a mouse ventilator (MiniVent 845, Hugo Sachs Elektronik, Hugstetten, Germany). These respiratory settings were found to result in optimal values of arterial pH, PO2, and PCO2 [1], [9], [17], [30], [62]–[66]. Body temperature was carefully monitored with a rectal probe and maintained as close as possible to 37.0°C. To prevent blood pressure drops, blood from a donor mouse was transfused at a dose of 40 mL/kg IV in three divided equal volume boluses. The chest was opened through a midline sternotomy with the aid of a dissecting microscope and a microcoagulator. An 8-0-nylon suture was passed under the mid-left anterior descending (LAD) coronary artery and a nontraumatic balloon occluder was applied on the artery. Ischemic PC was elicited by a sequence of six 4-min coronary occlusion (O)/4-min reperfusion (R) cycles (Figs. 1A, 1B and 1C). On day 2, mice were reanesthetized with sodium pentobarbital (60 mg/kg i.p.). The chest was reopened. The same 8-0-nylon suture and nontraumatic balloon occluder were used. Infarction was produced by a 30-min coronary occlusion and followed by 24 hours reperfusion (Figs. 1A, 1B and 1C). Ischemia was confirmed by noting ST elevation on ECG and blanching of the LV. After the coronary occlusion/reperfusion procedures, the chest was closed in layers and mice were allowed to recover [1], [9]–[11], [13]–[18], [20], [30], [40], [61]–[80].

**Table 2:** Size of left ventricle, risk region, and infarction in Phase I study.

Group	Age	Body	Heart	H/B	LV	RR	Infarct	RR	Infarct	Infarct
	(wk)	(g)	(mg)	(%)	(mg)	(mg)	(mg)	(% of LV)	(% of RR)	(% of LV)
Group I (COX-2^+/+^, non-PC, n = 13)	21.5±0.7	31.0±0.6	175±6	0.57±0.01	137±5	54±4	32±3	39.3±2.2	59.5±2.8	23.7±2.0
Group II (COX-2^+/+^, late PC, n = 15)	22.4±2.0	31.8±0.7	197±9	0.61±0.03	146±8	61±4	17±2^a^	41.1±2.1	27.2±2.6^a^	11.2±1.2^a^
Group III (COX-2^−/−^, non-PC, n = 11)	18.5±0.9	27.9±1.0	206±11^a^	0.74±0.03^ a^	166±11^a^	64±6	40±5	38.4±2.2	62.0±2.2	23.8±1.7
Group IV (COX-2^−/−^, late PC, n = 11)	22.6±1.1	29.5±0.4	197±10^a^	0.67±0.04^ a^	156±9^a^	61±5	40±2	39.2±2.4	59.8±2.0	23.2±2.0

Data are means ± SEM. BW, body weight; Heart, heart weight (ventricles and atria); H/B, ratio of heart weight to body weight; LV, left ventricular weight; RR, region at risk. There were no significant differences in the age, sex and weight of the mice among the 4 groups. Also, there were no significant differences in the region at risk among the 4 groups. ^a^ *P*<0.05 vs. group I.

**Table 3:** Size of left ventricle, risk region, and infarction in Phase II study.

Group	Age	Body	Heart	H/B	LV	RR	Infarct	RR	Infarct	Infarct
	(wk)	(g)	(mg)	(%)	(mg)	(mg)	(mg)	(% of LV)	(% of RR)	(% of LV)
V (B6, non-PC + naïve, n = 15)	12.1±1.3	30.7±0.8	156±6	0.51±0.02	117±5	47±4	29±2^_^	39.8±2.8	63.3±2.2^_^	25.0±1.7^_^
VI (B6, non-PC + vehicle, n = 6)	11.3±0.2	24.6±0.6	131±8	0.53±0.02	102±5	35±2	23±2^_^	34.9±3.2	65.7±3.9^_^	23.1±2.9^_^
VII (B6, non-PC + RO3244794, n = 8)	11.0±0.0	24.8±0.3	125±4	0.50±0.01	94±3	33±2	23±2	35.1±2.0	68.4±1.2^_^	24.2±1.7^_^
VIII (B6, late PC + naïve, n = 9)	12.6±2.0	29.6±1.0	155±6	0.53±0.02	112±4	42±2	14±2^a^	38.2±2.9	33.5±3.5^a^	33.5±3.5^a^
IX (B6, late PC + vehicle, n = 10)	9.8±0.4	24.3±0.6	126±4	0.52±0.01	93±3	35±3	11±2^a^	37.3±3.2	31.7±4.8^a^	11.5±1.9^a^
X (B6, late PC + RO3244794, n = 8)	9.4±0.2	25.3±0.4	137±8	0.55±0.03	99±6	34±3	22±3	34.5±1.3	63.8±4.5^_^	22.0±1.8^_^

Data are means ± SEM. BW, body weight; Heart, heart weight (ventricles and atria); H/B, ratio of heart weight to body weight; LV, left ventricular weight; RR, region at risk. There were no significant differences in the age, sex and weight of the mice among the 6 groups. Also, there were no significant differences in the region at risk among the 6 groups. ^a^ *P<0.05* vs. group V.

**Table 4:** Size of left ventricle, risk region, and infarction in Phase III study.

Group	Age	BW	Heart	H/B	LV	RR	Infarct	RR	Infarct	Infarct
	(wk)	(g)	(mg)	(%)	(mg)	(mg)	(mg)	(% of LV)	(% of RR)	(% of LV)
Group XI (IP^+/+^, non-PC, n = 16)	22.8±2.0	30.0±0.9	149±7	0.50±0.02	112±6	37±3	20±2	33.7±2.2	53.1±2.3	17.9±1.4
Group XII (IP^+/+^, late PC, n = 13)	22.8±2.9	29.4±0.8	140±8	0.47±0.02	106±7	41±3	15±1^a^	38.9±2.2	36.2±1.9^a^	13.8±0.8^a^
Group XIII (IP^−/−^, non-PC, n = 12)	19.8±2.5	30.3±1.0	162±7	0.54±0.02	125±5	40±3	21±2	32.0±2.3	52.9±2.1	16.9±1.4
Group XIV (IP^−/−^, late PC, n = 12)	22.4±2.0	29.6±0.8	142±6	0.48±0.01	106±5	37±3	19±2	34.9±2.0	52.4±3.7	18.1±2.8

Data are means ± SEM. BW, body weight; Heart, heart weight (ventricles and atria); H/B, ratio of heart weight to body weight; LV, left ventricular weight; RR, region at risk. There were no significant differences in the age, sex and weight of the mice among the 4 groups. Also, there were no significant differences in the region at risk among the 4 groups. ^a^ *P<0.05* vs. group XI.

**Table 5:** Rectal temperature and heart rate on the day of the 30-min coronary occlusion in Phase I study.

Group	Pre-occlusion	Occlusion			Reperfusion		
		5 min	15 min	30 min	5 min	10 min	15 min
***Temperature (°C)***							
Group I (COX-2^+/+^, non-PC, n = 13)	36.9±0.0	37.1±0.0	37.0±0.0	37.0±0.1	36.9±0.0	36.9±0.0	36.9±0.1
Group II (COX-2^+/+^, late PC, n = 15)	36.9±0.0	37.0±0.0	37.0±0.0	37.0±0.0	37.0±0.0	37.0±0.0	37.0±0.1
Group III (COX-2^−/−^, non-PC, n = 11)	36.9±0.0	37.1±0.0	37.0±0.0	37.0±0.1	37.0±0.1	37.0±0.1	37.0±0.1
Group IV (COX-2^−/−^, late PC, n = 11)	36.8±0.1	37.0±0.1	36.9±0.1	36.9±0.1	37.0±0.1	36.9±0.1	37.0±0.1
***Heart rate (beats/min)***							
Group I (COX-2^+/+^, non-PC, n = 13)	536±10	564±9	565±10	577±12	562±12	563±12	580±15
Group II (COX-2^+/+^, late PC, n = 15)	590±14	619±11	611±11	608±10	618±10	612±12	620±10
Group III (COX-2^−/−^, non-PC, n = 11)	549±13	576±13	567±11	579±14	579±13	573±9	584±11
Group IV (COX-2^−/−^, late PC, n = 11)	589±27	627±17	619±15	617±17	617±17	594±21	624±26

Data are means ± SEM. Measurements of rectal temperature and heart rate were taken before the 30-min coronary occlusion (pre-occlusion), at 5, 15 and 30 min into the 30-min occlusion, and at 5, 15 and 30 min after reperfusion. Rectal temperature was continuously monitored and carefully controlled throughout the experiment, as detailed in the text.

**Table 6:** Rectal temperature and heart rate on the day of the 30-min coronary occlusion in Phase II study.

Group	Pre-occlusion	Occlusion			Reperfusion		
		5 min	15 min	30 min	5 min	10 min	15 min
***Temperature (°C)***							
V (B6, non-PC + naïve, n = 15)	36.9±0.1	37.2±0.1	37.0±0.1	37.0±0.0	37.0±0.0	37.0±0.1	36.8±0.0
VI (B6, non-PC + vehicle, n = 6)	37.0±0.1	37.1±0.1	37.1±0.1	37.0±0.1	36.8±0.1	37.1±0.1	37.0±0.1
VII (B6, non-PC + RO3244794, n = 8)	37.0±0.1	37.1±0.0	37.0±0.1	37.0±0.1	36.9±0.0	37.0±0.1	37.1±0.1
VIII (B6, late PC naïve, n = 9)	37.0±0.1	37.2±0.1	36.9±0.1	37.1±0.1	36.9±0.1	36.9±0.0	36.9±0.1
IX (B6, late PC + vehicle, n = 10)	36.9±0.1	37.0±0.1	37.1±0.1	37.0±0.1	37.0±0.1	36.9±0.1	37.0±0.1
X (B6, late PC + RO3244794, n = 8)	36.9±0.1	37.2±0.1	37.0±0.1	37.0±0.1	37.0±0.1	37.0±0.1	36.9±0.1
***Heart rate (beats/min)***							
V (B6, non-PC + naïve, n = 15)	506±12	512±12	495±09	502±11	521±12	518±15	595±16
VI (B6, non-PC + vehicle, n = 6)	489±16	493±19	489±14	496±14	482±13	507±17	498±18
VII (B6, non-PC + RO3244794, n = 8)	566±09	618±17	620±16	636±15	640±16	634±21	635±21
VIII (B6, late PC + naïve, n = 9)	505±17	498±17	469±19	472±13	495±15	475±13	498±17
IX (B6, late PC + vehicle, n = 10)	566±09	618±17	620±16	636±15	640±16	634±21	635±21
X (B6, late PC + RO3244794, n = 8)	532±12	560±12	537±10	548±14	597±11	560±13	578±10

Data are means ± SEM. Measurements of rectal temperature and heart rate were taken before the 30-min coronary occlusion (pre-occlusion), at 5, 15 and 30 min into the 30-min occlusion, and at 5, 15 and 30 min after reperfusion. Rectal temperature was continuously monitored and carefully controlled throughout the experiment, as detailed in the text.

**Table 7:** Rectal temperature and heart rate on the day of the 30-min coronary occlusion in Phase III study.

Group	Pre-occlusion	Occlusion			Reperfusion		
		5 min	15 min	30 min	5 min	10 min	15 min
***Temperature (°C)***							
Group XI (IP^+/+^, non-PC, n = 16)	36.9±0.1	37.2±0.1	37.0±0.1	37.0±0.0	37.0±0.0	37.0±0.1	36.8±0.0
Group XII (IP^+/+^, late PC, n = 13)	36.9±0.1	37.2±0.1	37.1±0.1	37.0±0.1	37.1±0.1	37.1±0.1	37.1±0.1
Group XIII (IP^−/−^, non-PC, n = 12)	37.0±0.1	37.2±0.1	36.9±0.1	37.1±0.1	36.9±0.1	36.9±0.0	36.9±0.1
Group XIV (IP^−/−^, late PC, n = 12)	37.1±0.1	37.1±0.0	37.1±0.0	37.0±0.1	37.1±0.1	37.0±0.1	37.0±0.1
***Heart rate (beats/min)***							
Group XI (IP^+/+^, non-PC, n = 16)	506±12	512±12	495±9	502±11	521±12	518±15	595±16
Group XII (IP^+/+^, late PC, n = 13)	543±16	577±15	576±17	577±16	594±13	592±10	590±19
Group XIII (IP^−/−^, non-PC, n = 12)	505±17	498±17	469±19	472±13	495±15	475±13	498±17
Group XIV (IP^−/−^, late PC, n = 12)	536±13	569±16	553±16	529±24	555±17	563±19	567±19

Data are means ± SEM. Measurements of rectal temperature and heart rate were taken before the 30-min coronary occlusion (pre-occlusion), at 5, 15 and 30 min into the 30-min occlusion, and at 5, 15 and 30 min after reperfusion. Rectal temperature was continuously monitored and carefully controlled throughout the experiment, as detailed in the text.

### In vitro Tissue Staining

At the conclusion of the study, the heart was excised and perfused with Krebs-Henseleit solution through an aortic cannula. To delineate infarcted from viable myocardium, the heart was perfused with 1% TTC in phosphate buffer. To delineate the occluded/reperfused bed, the coronary artery was tied at the site of the previous occlusion and the aortic root was perfused with 10% phthalo blue dye. As a result of this procedure, the region at risk was identified by the absence of blue dye, whereas the rest of the LV was stained dark blue. The left ventricle was cut into 5–7 transverse slices, which were fixed in 10% neutral buffered formaldehyde, weighed, and photographed under a microscope [1], [9]–[11], [13]–[18], [20], [30], [40], [61]–[80].

### Infarct Size (IS) Measurement

Areas identified as infarct, at-risk, and nonischemic based on tissue staining were measured by computerized videoplanimetry and from these measurements infarct size was calculated as a percentage of the region at risk [1], [9]–[11], [13]–[18], [20], [30], [40], [61]–[80].

### Kidney and Liver Function Measurements

We collected the blood samples from the COX-2 knockout and wildtype mice before harvesting the mouse heart and sent to a commercial company to test the liver and renal function.

### Statistical Analysis

Data are reported as means ± SEM. Data analysis was performed using the SigmaStat software. Statistical comparisons were performed with one-way ANOVA followed by unpaired Student’s *t*-tests [9], [17], [30], [64].

**Table 8:** Liver profile of COX2 KO and WT mice.

	HEPATIC PROFILE										
Group	Age(wks)	BW(g)	ALT(U/L)	ALP(U/L)	Albumin(g/L)	Globulin(gm/dL)	AGRatio	AST(U/L)	BILI Total(mg/dl)	TP(gm/dL)	GGT(U/L)
**COX2-WT**	23.8±1.3	35.4±6.5	38.8±17.3	23.5±2.6	2.6±0.4	4.0±3.1	0.8±0.6	414.3±268.1	0.0±0.1	6.0±1.8	0.0±0.0
**COX2-KO**	22.5±2.4	27.7±1.9*	189.4±66.6*	43.8±15.4*	2.2±0.2	3.0±0.2	0.7±0.1	618.6±267.1	0.2±0.3	5.3±0.3	5.4±4.9

Age, age of mice (weeks); BW, body weight; ALT, Alanine Aminotransferase; ALP, Alkaline Phosphatase; AG RATIO, Albumin to Globulin Ration; AST, Aspartate Aminotransferase; BILI Total, Bilirubin Total; TP, Total Protein; and GGT, Gamma-Glutamyl Transferase. *P<0.05 vs. group COX-2 WT. Data = Mean ± SD.

**Table 9:** Renal profile of COX2 KO and WT mice.

	RENAL PROFILE							
Group	LDH(U/L)	BUN(mg/dl)	Creatinine(mg/dl)	Glucose(mg/dl)	Cl(mmol/L)	K(mmol/L)	Na(mmol/L)	Phosphorous(mg/dl)
**COX2-WT**	471.3±147.7	21.0±1.7	0.6±0.5	82.3±42.9	107.2±6.2	7.0±1.2	134.5±8.3	4.7±2.1
**COX2-KO**	1163.8±462.8*	98.0±24.4*	1.4±1.0	21.2±23.1	107.3±6.9	4.8±1.5*	145.1±1.1*	6.1±3.9

LDH, Lactate dehydrogenase; BUN, Blood Urea Nitrogen; Cl, Chloride; K, Potassium; Na, Sodium. *P<0.05 vs. group COX-2 WT. Data = Mean ± SD.

## Results

### Exclusions

A total of 211 mice were used for these experiments. Twenty-six mice died (Table 1); thus, total mortality was 12.3% (Table 1). Seventeen mice (8%) were excluded because of severe bleeding during surgery (2 mice), technical problem (12 mice, including malfunction of the ventilation system, damage to the coronary vessels, balloon malfunction) or inadequate postmortem staining (3 mice). One hundred and sixty-eight mice successfully completed the entire protocol and were included in the study (Table 1).

### General Characteristics, Heart Rate and Temperature

The mice used in the various groups had similar heart-to-body weight ratios. There were no significant differences in age, body weight, and risk region among groups (Tables 2, 3, and 4). Heart rate and rectal temperature before the 30-min coronary occlusion (pre-occlusion), at 5, 15 and 30 min into the occlusion, and at 5, 15 and 30 min after reperfusion in all groups are shown in Tables 5, 6, and 7. Heart rate, a fundamental physiological parameter that may impact infarct size, was similar in all the groups. Within the same group, heart rate did not differ significantly at any time-point before and during the 30-min occlusion or the ensuing reperfusion. By experimental design, rectal temperature, another potential determinant of infarct size, remained within a narrow physiologic range (36.8–37.2°C) in all groups (Tables 5, 6, and 7).

### Phase I: Role of *COX-2^−/−^* in Late PC *in vivo*

**Figure 2:**
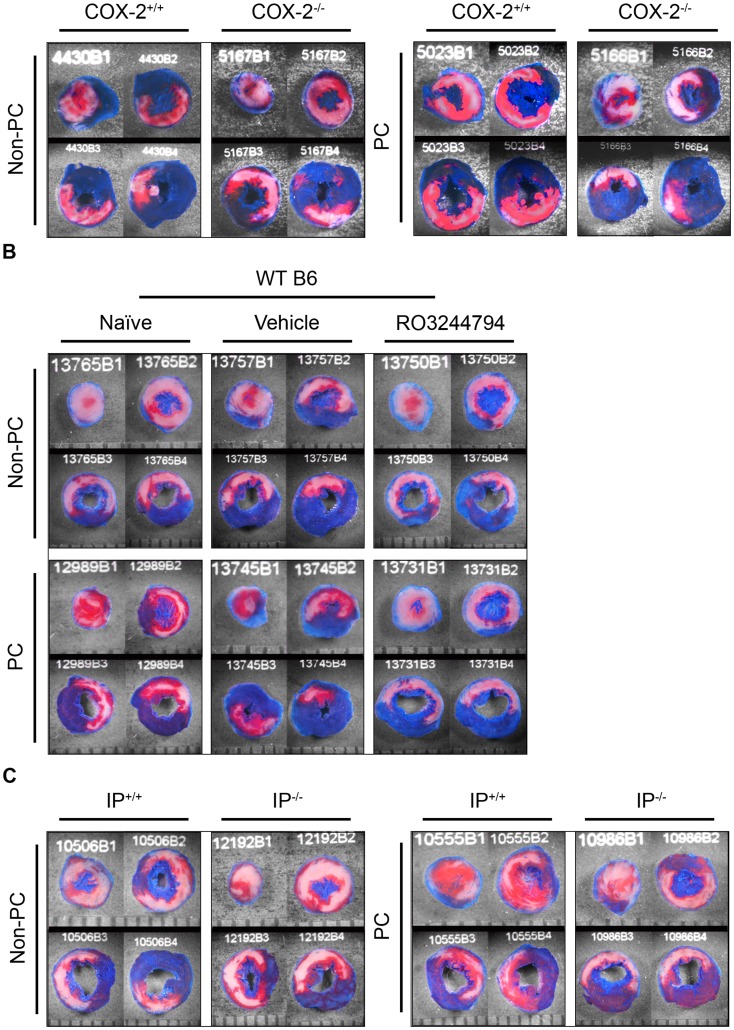
Representative examples of a heart from each group. The infarcted region was delineated by perfusing the aortic root with 2,3,5-triphenyltetrazolium chloride (TTC); the region at risk was delineated by perfusing the aortic root with phthalo blue after tying the previously occluded artery. As a result of this procedure, the nonischemic portion of the left ventricle (LV) was stained dark blue and viable tissue within the region at risk was stained bright red (TTC positive), whereas infarcted tissue was light yellow or white (TTC negative). **Phase I** (**panel A**). Non-preconditioned *COX-2^+/+^* and *COX-2^−/−^* mice have similarly large infarct sizes. PC 24 h prior to MI results in a significant reduction in infarct size in *COX-2^+/+^* but not *COX-2^−/−^* mice. **Phase II** (**panel B**). Non-preconditioned B6 mice in naïve, vehicle-treated, and RO3244794-treated groups have similar infarct sizes. PC results in significantly smaller infarct sizes in naïve and vehicle-treated mice but not in RO3244794-treated mice. **Phase III** (**panel C**). Non-preconditioned *IP^+/+^* and *IP^−/−^* mice have similar infarct sizes. PC results in a significant reduction in infarct size in *IP^+/+^* but not *IP^−/−^* mice. Scale at bottom is in mm. Note the large, confluent areas of infarction spanning most of the thickness of the LV wall, with thin rims of viable subendocardial tissue. This pattern was characteristic of all 7 nonpreconditioned groups (groups I, III, V–VII, XI and XIII) and all 3 PC groups (PC in *COX-2^−/−^* [group IV] and *IP^−/−^* mice [group XIV] or pretreated with the RO compound [group X]). In contrast, mice subjected to the PC protocol exhibited small, sporadic areas of infarction, a pattern that was characteristic of all 3 PC alone WT mice (groups II, VIII and XIII) and of the mice with PC + vehicle (group IX).

These studies were conducted in male mice, 19–23 wk old, weighing 28–32 g (Table 2). On day 1, mice were subjected to either the PC protocol or sham surgery. On day 2, all mice were subjected to a 30-min coronary occlusion and 24 h of reperfusion (Fig. 1A). Mortality was significantly greater in the KO group (Table 1), possibly because *COX-2^−/−^* mice suffered from renal and liver abnormalities (the data are shown in Tables 8 and 9). Heart slices demonstrating the postmortem staining of representative hearts for each group are shown in Figure 2A.

**Figure 3:**
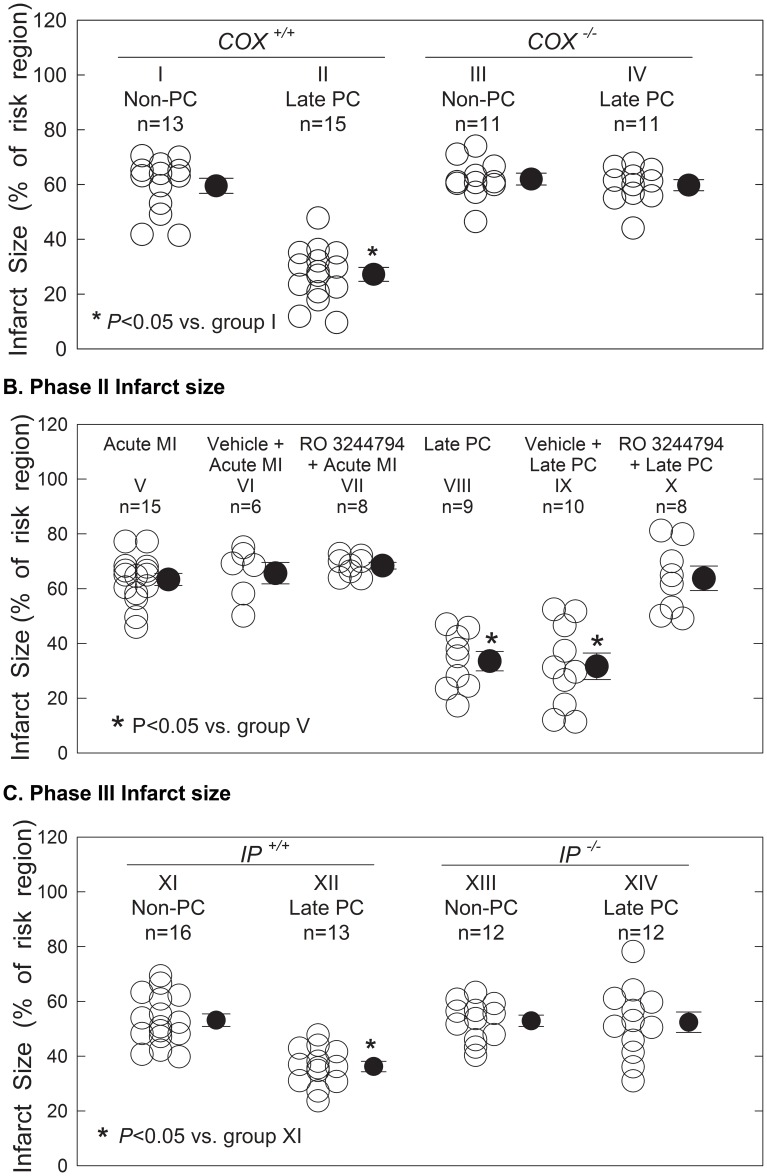
Myocardial infarct size in groups I–XIV. Infarct size is expressed as a percentage of the region at risk of infarction. Data are expressed as means ± SEM. **Phase I** (**panel A**). *COX-2^−/−^* mice did not exhibit the infarct-sparing effects of late PC. **Phase II** (**panel B**). RO3244794-treated mice did not exhibit the infarct-sparing effects of late PC. **Phase III** (**panel C**). *IP^−/−^* mice did not exhibit the infarct-sparing effects of late PC. (*) Marks a significant infarct size reduction in preconditioned mice compared with non-PC mice; *P*<0.05. ○, Individual mice; •, mean ± SE for respective group.

In non-PC *COX-2^+/+^* controls (Table 2 and Fig. 3A, group I), infarct size averaged 59.5±2.8% of the risk region. In PC *COX-2^+/+^* controls (Table 2 and Fig. 3A, group II), infarct size was significantly reduced to 34.0±3.7%; p<0.05, indicating the cardioprotective infarct-sparing effect conferred by late PC. In non-PC mice homozygous for a null COX-2 allele (COX-2^−/−^) (Table 2 and Fig. 3A, group III), infarct size (62.0±2.2%) was similar to *COX-2^+/+^* non-PC controls, indicating that COX-2 does not affect infarct size in the absence of PC. In contrast, *COX-2^−/−^* mice in the PC (Table 2 and Fig. 3A, group IV) group had a similar infarct size (59.8±3.0%) to non-PC *COX-2^+/+^* and *COX-2^−/−^* mice, indicating that deletion of COX-2 abolished the cardioprotection afforded by late PC. These results show that COX-2 does not affect infarct size in naïve conditions (no PC) and that targeted disruption of the COX-2 gene completely abrogates the infarct-sparing effect of late PC, providing unequivocal molecular genetic evidence for an obligatory role of COX-2 in late PC.

### Phase II: Role of IP in Late PC

The PGI2 receptor, IP, is known to be a specific transducer of PGI2 signaling in immunomodulation. We hypothesized that IP is a downstream mediator in COX-2 mediated late PC. We tested our hypothesis by inhibiting IP with the selective IP inhibitor RO3244794 and by using *IP^−/−^* mice.

**Figure 4:**
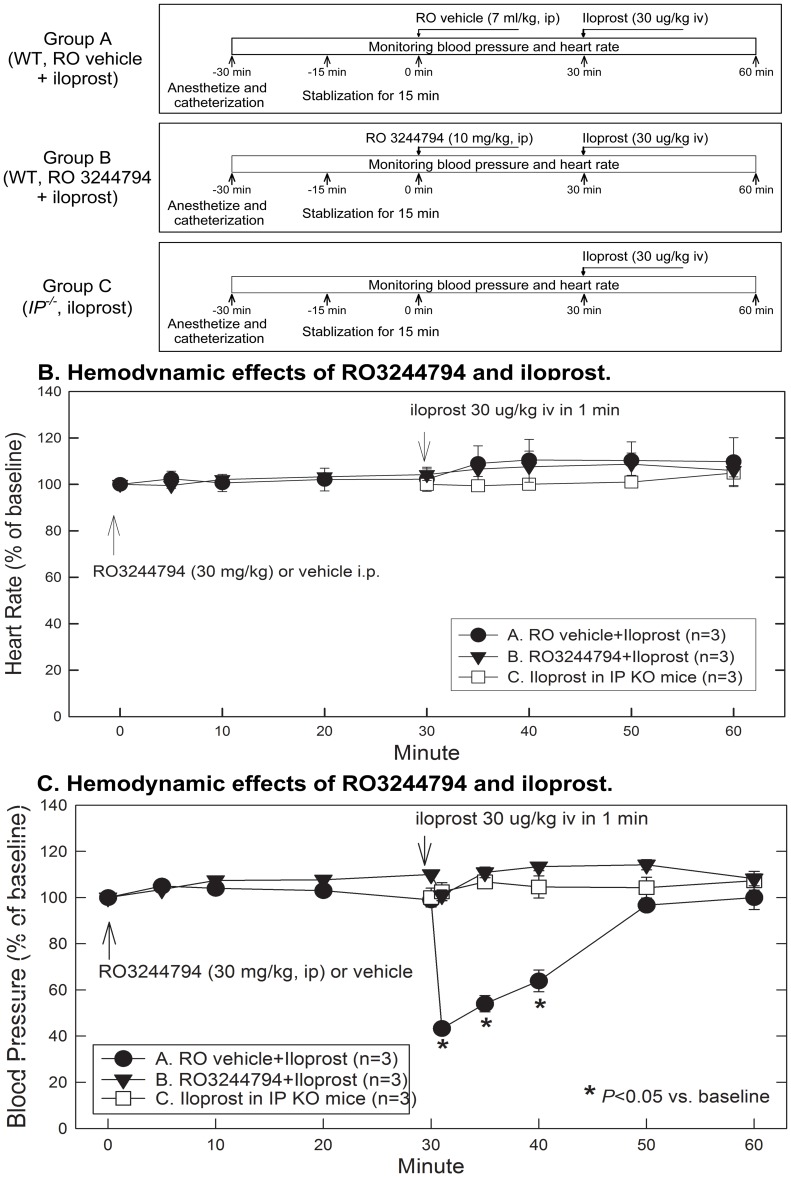
Pilot study. Effect of RO3244794 and *IP^−/−^* on iloprost-induced hypotension. Heart rate and mean arterial blood pressure (MAP) are shown as the changes of percentage of baselines in Figs. 4B and 4C, respectively. Data are expressed as means ± SEM. **A**) Experimental protocol for hemodynamic studies. **B**) Effect on heart rate (HR). There was no statistic significant difference in HR among the three groups (including the absolutely numbers). **C**) Effect on arterial blood pressure. Iloprost resulted in a significant drop in main arterial pressure (MAP); pretreatment with RO3244794 abolished the effect of iloprost on MAP, and iloprost had no effect on MAP in *IP^−/−^* mice.

### Pilot Studies

To confirm the specificity of this compound for IP receptors and to select the dose, we determined whether the specific IP antagonist (RO3244794) or IP deletion can attenuate the hypotensive effect induced by an IP agonist (iloprost).

Mice were assigned to three groups (Fig. 4A). Iloprost (a PGI2 analog) was administered intraperitoneally at a high dose of 30 µg/kg to *IP^+/+^* mice 30 min after RO3244794 (group A) or vehicle (group B). The same dose of iloprost was injected into *IP^−/−^* mice (group C). Iloprost injection to vehicle-pretreated animals resulted in a slight increase in heart rate (Fig. 4B) and a pronounced drop in mean arterial pressure (MAP, Fig. 4C), a normal response to iloprost. Administering iloprost to *IP^−/−^* mice did not affect the MAP. Similarly, in, RO3244794-treated mice, iloprost failed to reduce MAP. These data indicate that RO3244794, at the doses used here, effectively inhibits the PGI2 effect on MAP. RO3244794 did not alter baseline MAP and heart rate, indicating that the drug in the doses used does not have significant hemodynamic side effects.

### Selective IP Inhibition with RO3244974 Abolishes the Infarct-sparing Effect of Late PC *in vivo*

Male C57BL/6J (B6) mice, 9–13wk old; weighing 24–31 g, were used to test whether selective pharmacological inhibition of IP abrogates late PC. RO3244794 (10 mg/kg) or vehicle (7 ml/kg) was administered intraperitoneally 30 min before the 30-min occlusion. Representative examples of postmortem staining are shown in Figure 2B.

In non-preconditioned untreated controls (Table 3 and Fig. 3B, group V), infarct size averaged 63.3±2.2% of the risk region. In preconditioned untreated controls (Fig. 3B, group VIII), infarct size was significantly reduced to 33.5±3.5% (p<0.05), indicating the cardioprotective infarct-sparing effect conferred by late PC. In non-preconditioned mice treated with the selective IP inhibitor RO3244794 (Table 3 and Fig. 3B, group VII), infarct size (68.4±1.2%) was similar to untreated non-preconditioned controls, indicating that IP does not confer cardioprotective effects in the absence of PC. In preconditioned mice treated with RO3244794 (Table 3 and Fig. 3B, group X), infarct size (63.8±4.5%) was similar to non-preconditioned untreated controls and RO3244794-treated mice, indicating that inhibition of IP abolishes the cardioprotection offered by late PC. To determine whether the RO3244794 vehicle (0.2 M Trizma base) had any biological effects, non-preconditioned and preconditioned mice were treated with vehicle in the same amount as required for RO3244794 delivery. The infarct size of non-preconditioned vehicle-treated mice (65.7±3.2%; Table 3 and Fig. 3B, group VI) was very similar to non-preconditioned untreated controls (group V). In contrast, treating preconditioned mice with vehicle (Table 3 and Figure 3B, group IX) resulted in a significant reduction in infarct size (32.3±4.5%; p<0.05) comparable to that seen in preconditioned untreated mice (group VIII). These results indicate that selective IP inhibition by RO3244794 results in abolition of the infarct-sparing effect of late PC, implying a prominent role of IP in transducing the signals mediating late PC.

### Phase III: Deletion of IP Blocks the Cardioprotective Infarct-sparing Effect of Late PC *in vivo*

To corroborate the pharmacologic studies in phase II, in phase III we performed studies using genetic ablation of IP. We tested if targeted disruption of the IP gene abrogates late PC in male mice, 20–21 wks old; weighing 25–30 g. Mortality was not significantly different among the four groups. Representative examples of postmortem staining are shown in Figure 2C.

In non-preconditioned *IP^+/+^* controls (Table 4 and Fig. 3C, group XI), infarct size averaged 50.7±2.7% of the risk region. In preconditioned *IP^+/+^* controls (Table 4 and Fig. 3C, group XII), infarct size was markedly reduced to 38.9±2.6% (p<0.05). In non-preconditioned mice homozygous for the null IP allele (*IP^−/−^*) (Table 4 and Fig. 3C, group XIII), infarct size (52.9±2.1%) was similar to *IP^+/+^* non-preconditioned controls, confirming that IP does not confer cardioprotective effects in the absence of PC. In contrast, when *IP^−/−^* mice were preconditioned (Table 4 and Fig. 3C, group XIV), infarct size (52.4±3.7) was similar to non-preconditioned *IP^+/+^* and *IP^−/−^* mice. These results indicate that the IP receptor does not modulate myocardial ischemia/reperfusion injury at baseline and that targeted disruption of the IP gene completely abrogates the infarct-sparing effect of late PC, providing, for the first time, molecular genetic evidence for an obligatory role of IP in the cardioprotection conferred by late PC.

## Discussion

Over the last 20 years, considerable efforts have been directed towards better understanding of the molecular interplay involved in the process of PC. The cardioprotective effects of PC are manifest in two phases [7], [81]–[83], an early phase starting few minutes after the ischemic stimulus lasting for 2–4 h and a late phase starting about 12–24 h after the stimulus and lasting for 24–72 h [7], [81]–[83]. The late phase of PC is mediated by pathways involving modulation of gene transcription, producing relatively long lasting effects [6], [7], [24], [81]. A number of candidate genes have been identified that can mediate this long lasting late phase of PC [7], [9], [26], [60], [84]–[86]. Understanding the molecular basis of PC may provide targets for developing drugs that can reproduce the cardioprotective effects conferred by the late phase of PC with minimal side effects.

Clinical evidence of increased cardiovascular mortality following use of COX-2 inhibitors has brought COX-2 into focus as a cardioprotective molecule [87]–[94]; however, even before this evidence started to appear, we showed for the first time the cardioprotective effects of COX-2 and its involvement in the late phase of PC [29], [30], [33], [35]–[37], [40]. We demonstrated upregulation of cardiac COX-2 mRNA/protein and PGE_2_/6-keto-PGF_1α_ levels in a rabbit model [35] and a mouse model [32] of late PC. We further demonstrated that the infarct-sparing effect of late PC was abolished by COX-2 inhibitors (NS-398 and celecoxib) administered 24 h after PC [29], [30]. Thus far, the experimental evidence supporting the role of COX-2 in late PC has been based on the observations that: 1) COX-2 and prostanoids are upregulated in animal models in which the infarct-sparing effects of late PC are evident [29], [32] and, 2) pharmacologic COX-2 inhibitors abolish late PC [29], [30]. These data are limited by the possible nonspecific effects of COX-2 inhibitors. Therefore, in this study, we have assessed the role of COX-2 in late PC by using *COX-2^−/−^* mice. The abrogation of late PC in *COX-2^−/−^* mice provides conclusive, unequivocal proof of the role of COX-2 in mediating the late phase of PC.

*COX-2^−/−^* mice may have poor survival secondary to the key role played by COX-2 in maintenance of hemodynamics, immunity and other vital functions. Understanding the molecules downstream of COX-2 is important if this pathway is to be exploited for therapeutic purposes. Although it appears that COX-2 probably mediates its cardioprotective effects via upregulation of PGI2 and/or PGE_2_ [29], the downstream signal transduction pathways mediating late PC via COX-2-derived prostanoids are unknown. Studies have pointed to prostacyclin (PGI2) [36], [71] and PGE2 [71] as the main prostanoids involved in cardioprotective effects during ischemia/reperfusion myocardial injury. A previous study from our group has shown that 6-keto-PGF_1α,_ a stable metabolite of PGI2, is upregulated in opioid-induced late phase PC [41]. In the same study it was shown that COX-2 inhibition resulted in abolition of the infarct-sparing effect of opioid-induced late PC. This study suggests that coupling of COX-2 and PGI2 is the most likely mechanism mediating the cardioprotective effects of late PC. Given this evidence, we hypothesized that the PGI2 receptor, IP, is a key mediator, downstream from COX-2/prostanoids, of the late phase PC. Our experiments show that late PC was abolished by selective IP inhibition by RO3244794 and that *IP^−/−^* mice lack the infarct-sparing effect of late PC. This is the first study to establish the obligatory role of IP as a mediator of late PC.

In the Phase I study, there was no significant difference in infarct size in non-preconditioned *COX-2^−/−^* mice compared with non-preconditioned *COX-2^+/+^* mice, indicating that COX-2-dependent signaling does not modulate ischemia-reperfusion injury in the basal (non-preconditioned) state (Table 2 and Fig. 3A). The result is internally consistent and corroborated with our previous findings which we tested the effect on the infarct size with COX-2 inhibitors in naïve rabbits [29] and mice [30] in vivo. Although, this result is contrary to that of Camitta et al (Circulation 2001), who reported that *COX-2^−/−^* mice exhibited a significantly larger infarct size compared to *COX-2^+/+^* [95]. We think that: 1) the models were different (Langendorff setting vs. in vivo) between these two studies; 2) the duration of LAD occlusion in the Camitta study was shorter (20 min vs. 30 min) than our study; 3) the duration of reperfusion in the Camitta study was also shorter (40 min vs. 24 hours) than our study. It is possible that COX-2 signaling may play different role in modulating injury with different durations of ischemia and reperfusion. In the Phase III study, there was no significant difference in infarct size in non-preconditioned *IP^−/−^* mice compared with non-preconditioned *IP^+/+^* mice, indicating that IP-dependent signaling does not modulate ischemia-reperfusion injury in the basal (non-preconditioned) state (Table 4 and Fig. 3C). The same result was also confirmed in the phase II study of pretreatment of IP antagonist, RO3244794 in the naïve mice (Table 3 and Fig. 3B). This result is contrary to that of Xiao et al (Circulation 2001), who reported that *IP^−/−^* mice exhibited a significantly larger infarct size compared to *IP^+/+^*. We do not have an obvious explanation for this discrepancy; however, the duration of LAD occlusion in the Xiao study was longer (60 min vs. 30 min) than our study. It is possible that IP signaling may become important in modulating injury with longer durations of ischemia.

The combination of pharmacological and genetic evidence strongly supports our hypothesis that IP is a key downstream molecular mediator of late PC in the COX-2/prostanoid pathway. Additionally, our lab and other investigators have shown that the transcription factor STAT3 plays a key role in late PC by upregulating the expression of cardioprotective proteins such as iNOS, COX-2, HO1, and anti-apoptotic factors [7], [42], [96]. Recent studies in human erythroleukemia cells have shown that IP mediates STAT3 activation by stimulating STAT3 Tyr(705) and Ser(727) phosphorylation [97]. Thus, it appears that IP not only mediates signal transduction for COX-2 but also may act as a facilitator for feedback enhancement of multiple pathways mediating the late phase of PC. This receptor is therefore emerging as an important player in the pathophysiology of late PC.

The prostanoid receptors are a family of cell surface 7-transmembrane-domain G-protein coupled receptor (GPCR) classified into five subtypes [98]. The human IP receptor stimulates downstream activation primarily coupled to Gα_s_-adenylyl cyclase but also has been shown to act through Gq-mediated phospholipase C (PLC) activation [97]. We currently have a good understanding of the structure of IP based on homology modeling with the thromboxane A2 (TP) receptor and the cellular processing of IP from transcription to trafficking [99]. The already existing structural [100], [101] and biochemical knowledge of IP should facilitate strategies for pharmacological modulation of IP for therapeutic purposes.

Identifying selective and specific IP agonists would be an appealing pharmacological approach to mimic the late phase of PC. For example, targeted drug screening strategies may lead to the discovery of selective IP agonists that could mimic the cardioprotective effects of late PC.

In conclusion, the present results advance our understanding of the intricate process of late PC. To the best of our knowledge, this is the first study to demonstrate the obligatory role of COX-2 in late PC by using a genetic approach. This is also the first study to demonstrate, using genetic and pharmacological evidence, the obligatory role of IP in this process. Finally, we have shown that selective IP modulation for cardioprotection is feasible, suggesting that it has the potential to be exploited as a therapeutic target.
